# Stress Fractures among Paramilitary Trainee Visiting a Paramilitary Hospital of Nepal: A Descriptive Cross-sectional Study

**DOI:** 10.31729/jnma.7268

**Published:** 2022-01-31

**Authors:** Sailendra Kumar Duwal Shrestha, Ajaya Basnet, Netra Bahadur Karki, Prabin Nepal, Umash Karki, Samir KC, Kumar Shrestha, Basanta Tamang, Mahendra Raj Shrestha

**Affiliations:** 1 Department of Orthopedic and Trauma Services, Nepal Armed Police Force Hospital, Kathmandu, Bagmati, Nepal; 2 Department of Medical Microbiology, Shi-Gan International College of Science and Technology, Tribhuvan University, Kathmandu, Bagmati, Nepal; 3 Department of Clinical Laboratory, Nepal Armed Police Force Hospital, Kathmandu, Bagmati, Nepal

**Keywords:** *bone mineral density*, *Nepal*, *stress fractures*, *vitamin D*

## Abstract

**Introduction:**

Young paramilitary recruits, who undergo strenuous exercise during basic training, are often presented with stress fractures, which could be due to an inadequate vitamin D (25-hydroxyvitamin D) intake. This study aimed to find the prevalence of stress fracture among young paramilitary trainees visiting the orthopedic outpatient department of a paramilitary hospital.

**Methods:**

This was a descriptive cross-sectional study done among paramilitary trainees in a paramilitary Hospital of Nepal between April 2019 to April 2021. The study was approved by the Ethical Review Board (Reference number: 1003) of the Nepal Health Research Council. Convenience sampling was used. Anthropometric variables, serum 25-hydroxyvitamin D level, and bone mineral density of spine and hip were determined. Data analysis was performed using the Statistical Package for the Social Sciences software version 17.0. Point estimate at 95% Confidence Interval was calculated along with frequency, proportion for binary data and mean, standard deviation for continuous data.

**Results:**

Among 417 young paramilitary trainees, 24 (5.76%) (3.52-7.99 at 95% Confidence Interval) were found to have a stress fracture. The stress fracture patients had a serum 25-hydroxyvitamin D level of 21.47ng/mL±6.98. Similarly, the bone mineral density value of the spine and hip among the patients was -1.34g/cm2±1.37 and 0.36g/cm2±1.24, respectively.

**Conclusions:**

The prevalence of stress fracture among young paramilitary trainee was high compared to previous studies. Additionally, average Vitamin D and the bone mineral density value of the spine and the total hip among stressed fractured patients were also low.

## INTRODUCTION

Stress fractures are particularly a common type of bone injury, which occurs either as a result of excessive stress on the bone,^1^ exceeding the bone's capacity to withstand and heal from those forces,^2^ or as insufficiency fractures due to secondary osteoporosis.^1^ Such fractures act as a source of significant morbidity in physically active people, from recreational exercisers to military recruits.^1,2^

The paramilitary trainee represents a unique population exposed to intense physical stress,^3^ characterized by excessive stress on the bone.^1^ Energy deficits, due to restrictive dietary patterns, in such personnel may reduce the muscle mass and bone strength needed for optimal performance, which makes them more susceptible to developing stress fractures.^1,4^ Such fractures require a varying recovery period and are often accompanied by the possibility of failure to return to military duty.^5^

Therefore, this study aimed to find the prevalence of stress fractures among paramilitary trainees in a paramilitary hospital of Nepal.

## METHODS

A descriptive cross-sectional study was done among young paramilitary trainees who presented to Nepal Army Police Force Hospital (NAPFH) Balambu, Kathmandu, Nepal, between April 2019 to April 2021. The study was approved by the Ethical Review Board (Reference number: 1003) of the Nepal Health Research Council, Ramshah Path, Kathmandu, Nepal. All the paramilitary trainees who attended the orthopedic department of NAPFH were included in the study. Patients using the medications, such as oral steroids, bisphosphonates, and lithium, known to affect bone health were excluded. Convenience sampling was done. The sample size was calculated using the following formula:

n = Z^2^ × (p × q) / e^2^

  = (1.96)^2^ × 0.50 (1-0.50) / (0.05)^2^

  = 385

Where,

n= required sample size
Z= 1.96 at 95% of Confidence Interval (CI)
p= prevalence of stress fracture among young paramilitary trainees visiting the orthopedic outpatient department of a paramilitary hospital taken as 50% for maximum sample size
q= 1-p
e= margin of error, 5%

The calculated sample size was 385. However, a sample size of 417 meeting the selection criteria were taken.

All patients who were initially suspected of stress fracture were sent for plain film X-ray for a confirmatory diagnosis. The orthopedic surgeon treating the recruits recorded injuries and confirmed the diagnosis of a stress fracture after a review of the images based on an X-ray.

Approximately 5ml of venous blood samples were drawn from antecubital venipuncture into tubes (BD Vacutainer; Becton, Dickinson and Company®, 2002 BD). The samples were immediately processed for the determination of serum 25(OH)D concentration and if any case of delay, samples were analyzed within 4 hours at the clinical laboratory of biochemistry of NAPFH.

Vitamin D estimation was performed in the clinical laboratory of biochemistry by iCHROMA^TM^II (Boditech, Korea). A value <10ng/ml was considered deficient, 10-30ng/ml was considered insufficient, and above 30ng/ml was considered sufficient.^6^

Bone mineral density (g/cm^2^) was measured by dualenergy x-ray absorptiometry (Encore V18, Prodigy, Lunar, GE Healthcare, United States) in the lumbar spine (LS) and the total hip (TH) at baseline.

Anthropometric measurements included body weight, height, and calculation of body mass index (BMI). Height (±1cm) was measured using a wall-mounted stadiometer. Weight (±100gm) was measured on a calibrated electronic scale. BMI was calculated as mass (kg) divided by height squared (m^2^).

Data analysis was performed using the Statistical Package for the Social Sciences software version 17.0. Data analysis of the age, sex, height, weight, and BMI were conducted by descriptive statistics including mean and standard deviation. Point estimate at 95% Confidence Interval was calculated along with frequency, proportion for binary data, and mean, standard deviation for continuous data.

## RESULTS

Among 417 young paramilitary trainees, the stress fracture was found in 24 (5.76%) (3.52-7.99 at 95% Confidence Interval) trainees. There were 19 (79.17%) males and 5 (20.83%) females among stress fracture patients. The patients had a median age of 20 years (Interquartile Range: 19.25-21). The BMI of stress fracture patients was 20.92kg/m^2^±3.06 (Table 1).

**Table 1:** Sociodemographic characteristics of young paramilitary trainees with stress fracture (n= 24).

Variables	
Age (Years)	n (%)
19	6 (25)
20	7 (29.17)
21	8 (33.33)
22	2 (8.33)
23	1 (4.17)
**Gender**	
Male	19 (79.17)
Female	5 (20.83)
**Mean±SD**	
Height (m)	1.66±0.08
Weight (kg)	57.50±7.10
BMI (kg/m^2^)	20.92±3.06

* IQR: Interquartile Range

† SD: Standard Deviation

Among the trainees with stress fractures, fracture in the femur was most common 14 (58.33%), followed by a fracture in the tibial shaft 7 (29.17%), pubic rami 2 (8.33%), and tibia fibula combination 1 (4.17%) (Table 2).

**Table 2:** Location of stress fractures (n= 24).

Location	n (%)
Femur	14 (58.33)
Tibial shaft	7 (29.17)
Pubic rami	2 (8.33)
Tibia fibula combination	1 (4.17)

The mean (±SD) serum 25(OH)D level of stress fracture patients was 21.47ng/ml±6.98. The stress fractured patients with insufficient concentrations (10-30ng/ml) of 25(OH)D were predominant 20 (83.33%), followed by the patients with sufficient concentrations (30-100ng/ml) 3 (12.5%), and deficient concentration 1 (4.17%) of 25(OH)D. The stress fractured patients had BMD values of the spine and total hip as -1.34g/cm^2^±1.37 and 0.36g/cm^2^ ±1.24, respectively (Table 3).

**Table 3:** Status of vitamin D leveland Bone mineraldensity among young paramilitary trainees with stressfracture (n= 24).

Characteristics	n (%)
**25(OH)D level (ng/ml)**	
Deficient	1 (4.17)
Insufficient	20 (83.33)
Sufficient	3 (12.5)
**Bone mineral density (g/cm^2^)**	
Spine	-1.34±1.37
Hip	0.36±1.24

## DISCUSSION

An adequate level of serum 25(OH)D is essential for maintaining normal physiological function linked to skeletal and non-skeletal tissue as well as for maintaining calcium and phosphate levels,^7^ which ultimately affects different cellular functions like bone mineralization, muscle contraction, nerve conduction, and enzymatic functions.^8^ Despite the known pleiotropic functions of 25(OH)D, its deficiency and its association with the occurrence of stress fractures in military trainees has become a significant issue, as previously documented by several studies.^4^-^7–9,10^

Military training is characterized by a major change in nutritional habits, partially resulting from eating in mess and rations provided in the field,^3^ which makes such personnel more prone to suffer from recurrent stress fractures.^5^ This study showed that individuals from the paramilitary force with male sex (79.17%) and the median age of 20 years (29.17%) were at a higher risk of stress fractures, which concedes with the conclusion of Magar, et al, who reported similar findings of male sex (88.88%) and the age of (20.7 years).^11^

Previously, several studies have correlated an increased risk of stress fractures in paramilitary trainees with the lower levels of serum 25(OH) D.^7,9,12^ In this study, 25(OH)D level in stress fractured patients (21.47ng/ml±6.98) was lower. This finding was consistent with a study conducted on 800 Finnish military recruits, which reported that a lower level of 25(OH)D (<75.8nmol/L) was a significant risk factor for a stress fracture.^12^ As discussed earlier, such a low level of 25(OH)D in paramilitary trainees could be due to a change in lifestyle, less exposure to sunlight, and physical and mental stress during the training.^5^

Various studies had shown a decrease in the bone mineral density of a weight-bearing bone at a site where stress fractures occur, especially in individuals under strenuous physical training.^13,14^ This study found that trainees with stress fractures had lower bone mineral densities, more commonly in the femoral head (0.36±1.24)gm/cm^2^ and the lumbar spine (-1.34±1.37) gm/cm^2^. Similarly, a case-control study in athletes, matched for anthropometric details and exercise history found lowered BMD of the spine and proximal femur sites in the stress fractured athletes compared with uninjured control athletes.^15^ Simultaneously, Cashman, et al. reported that lower 25(OH)D status was associated with the lower bone mineral density of the forearm in a study of 12- and 15-year-old girls.^14^ Another case-control study, involving 600 Navy recruits showed a twofold increased risk of stress fractures of the tibia and fibula in recruits with a 25(OH)D level of <20ng/mL as compared to the recruits with concentrations >40ng/mL and significantly correlated that lower bone mineral density with lower serum 25(OH)D level.^17^ These analyses were similar to our finding, specifically in context to lowered serum 25(OH)D level directly proportional to lower BMD of the spine.

This was a cross-sectional study and data were collected retrospectively among young trainees who seek medical care. The findings of this study are based on a limited number of observational studies; therefore, further well-designed higher studies with larger sample sizes are necessary to understand the significance of serum 25(OH)D level and BMD for the mitigation of stress fractures in paramilitary trainees.

## CONCLUSIONS

The prevalence of stress fracture among young paramilitary trainees was high as compared to previous studies. Similarly, the BMD value of the spine and the total hip and the average Vitamin D level among these patients were also low. If such lowered values of these bone parameters can be detected early, prompt management can be carried out and thus complications can be prevented.
